# Detoxification of parthenium (*Parthenium hysterophorus*) and its metamorphosis into an organic fertilizer and biopesticide

**DOI:** 10.1186/s40643-017-0156-6

**Published:** 2017-06-15

**Authors:** Naseer Hussain, Tasneem Abbasi, Shahid Abbas Abbasi

**Affiliations:** 1 Centre for Pollution Control & Environmental Engineering, Pondicherry University, Chinakalapet, Puducherry, 605 014 India; 2 Department of Fire Protection Engineering, Worcester Polytechnic Institute, Worcester, MA 01609 USA

**Keywords:** Invasive weed, Allelopathy, Organic fertilizer, Biopesticide, Vermicomposting

## Abstract

**Background:**

Vermicompost of the toxic and allelopathic weed parthenium (*Parthenium hysterophorus*) was explored for its possible use as an organic fertilizer. Replicated plant growth trials were conducted using four levels of parthenium vermicompost (0, 2.5, 3.75, and 5 t/ha) to assess their effects on the germination, growth, and fruition of a typical food plant ladies finger (*Abelmoschus esculentus*). Additionally the role of vermicompost in reducing plant pests and disease was evaluated.

**Results:**

Vermicompost encouraged the germination and growth of ladies finger at all levels of vermicompost application, with best results obtained in 5 t/ha treatments. The positive impact extended up to the fruit yield. Vermicompost application also improved the quality of fruits in terms of mineral, protein, and carbohydrate contents, and reduced the disease incidence and pest attacks.

**Conclusions:**

The studies establish the fact that parthenium acquires all the qualities of a good organic fertilizer with concomitant loss of its toxic and allelopathic properties after it gets vermicomposted. The findings raise the prospects of economical and eco-friendly utilization of billions of tons of parthenium biomass which is generated annually but goes to waste at present.

## Background

*Parthenium hysterophorus*, commonly called as congress grass, is among the world’s seven most noxious and devastating weeds (Patel 2011). It is an annual flowering, erect, and severally branched ubiquitous herb, which grows aggressively in a wide range of habitats (Akter and Zuberi 2009). Due to the absence of any effective natural enemies and due to its allelopathic nature, large seed bank, and fast growth rate, parthenium grows luxuriantly all through the year, infesting millions of hectares of land masses including agricultural fields, parks, orchards, railway tracks, and other open areas (Wiesner et al. 2007; Nigatu et al. 2010; Qureshi et al. 2014). This proves disastrous in terms of monopolizing of space and nutrients by parthenium at the expense of other vegetation, consequent loss of biodiversity, and associated ecological imbalances (Hussain et al. 2016a, b). Parthenium’s dominance over other vegetation is fostered by the presence of allelopathic compounds in parthenium, especially parthenin, hysterin, ambrosin, and flavonoids (Maishi et al. 1998; Khan and Abbasi 1998; Knox et al. 2011; Patel 2011; Kaur et al. 2014). These compounds are leached when dew or rain falls on parthenium (Abbasi and Abbasi 2011), and reach the underlying soil. There they cause toxicity, discouraging the growth of other vegetation in the vicinity of parthenium, thereby aiding and abetting the spread of parthenium monocultures (Hussain et al. 2016a, b).

Parthenium has mammalian toxicity as well. It causes dermatitis, eczema, asthma, allergic rhinitis, hay fever, black spots, burning, and blisters around eyes in mammals, including humans (Gunaseelan 1987; Towers and Rao 1992; Maishi et al. 1998; Morin et al. 2009; Akhtar et al. 2010). Exposure to parthenium also causes systemic toxicity including loss of skin pigmentation, dermatitis, diarrhea, and degenerative changes in liver and kidneys in livestock who accidentally graze upon parthenium (Gunaseelan 1987; Rajkumar et al. 1988; Lakshmi and Srinivas 2007).

The eradication of parthenium is a major challenge, primarily because of its epidemic proliferation, strong reproductive potential, hardiness, and competitiveness, apart from its wide ecological adoptability. Efforts made across the world to find persistent methods of controlling parthenium by way of mechanical, chemical, or biological means, have at best achieved only partial and temporary success (Manoj 2014; Hussain et al. 2016a). The weed has never been eradicated from any country and its spread in all the tropical and sub-tropical regions of the world is only increasing with time. When viewed as a resource, for the generation of green manure, biogas production, biopesticides, and drugs (Abbasi et al. 1990; Kishor et al. 2010; Kumar et al. 2012; Gunaseelan 1987; Patel 2011; Tauseef et al. 2013; Singh and Garg 2014; Kumar et al. 2014; Anwar et al. 2015; Hussain et al. 2016b), parthenium has proved uneconomical, expensive, and unsustainable. Thus finding an ecologically sound and economically viable means by which parthenium can be gainfully utilized in large quantities appears to be the only recourse which can make it profitable to regularly harvest the weed, thereby keeping it under some control.

One such option is conversion of parthenium biomass into organic fertilizer through vermicomposting. When a substrate is vermicomposted, it converts the latter into fine peat-like material and transforms some of its nutrients into more bioavailable forms (Hussain et al. 2015, 2016a, b. The vermicompost acquires several species of microflora, besides hormones and enzymes as it passes though the earthworms' gut (Pramanik et al. 2007; Ievinsh 2011). Past studies have reported that vermicompost derived from animal manure stimulated seed germination (Atiyeh et al. 2000; Zaller 2007; Lazcano et al. 2010), enhanced plant growth (Edwards Clive 2004; Lazcano et al. 2009; Samrot et al. 2015), and the yield and the quality of fruits (Singh et al. 2008; Doan et al. 2015) of several plant species. They are also believed to induce resistance in plants against pests and disease (Yardim et al. 2006; Edwards et al. 2010; Serfoji et al. 2010; Carr and Nelson 2014). But it is not yet established whether vermicompost derived from plants, more so from toxic and allelopathic plants like parthenium, can be as benign and effective an organic fertilizer as manure-derived vermicomposts are. In our recent studies (Gajalakshmi and Abbasi 2002; Hussain et al. 2016a, b) it was seen that soil augmented with parthenium vermicompost had enabled better germination success of four species of food plants compared to the control soil. But will the beneficial effect extend to plant growth, fruit yield, and quality of the fruit? Will the parthenium vermicompost also help the fertilized plants to repel pathogens the way manure-derived vermicomposts are known to do? In order to find definite answers to these questions, the present field-scale study has been carried out on the effect of parthenium vermicompost on a common vegetable ladies finger (*Abelmoschus esculentus*) from germination stage right up to the quality and yield of fruit.

## Experimental

The experiment was carried out at the Pondicherry University, Puducherry, India, which is located along the eastern coast of the South India (11° 56′ N, 79° 53′ E). This region experiences hot summers, with maximum day temperature 35–38 °C, during March–July and mild winters during December–February (maximum day temperature 29–32 °C). The average annual rainfall is about 1300 mm, concentrated mainly during October–December but with a few rainy days occurring in July–August and January as well. The study was conducted during February–May which is ideal for growing ladies finger in the study area (ICAR 2011). The vermicompost used in the experiment was produced from parthenium leaves, which were collected from the vicinity of Pondicherry University campus. The leaves were washed to remove the adhering soil and subjected to the earthworm species *Eisenia foetida* in pulse-fed, high-rate vermireactors, as detailed by Nayeem-Shah (2014), and Nayeem-Shah et al. (2015). There was no pre-composting or any manure supplementation. The vermicast was periodically harvested in each pulse and this precisely quantifiable product of the earthworm action was deemed as vermicompost (Abbasi et al. 2009, 2015). The characteristics of the vermicompost are detailed earlier by Hussain et al. (2016a, b).

To study the effect of parthenium vermicompost on several stages of plant growth, an outdoor experiment was conducted using low-density polyethylene (LDPE) bags of 50-l capacity as containers of the soil. In separate treatments, vermicompost was supplemented in bags to the extent of 0, 2.5, 3.75, and 5 t/ha. For each treatment 35 bags were set and in each bag 5 ladies finger *(Abelmoschus esculentus*) seeds were sown. Soil used in the study was not used for cultivation in the past and had not received any anthropogenic input of fertilizers. Germination success was assessed up to 8 days and has been presented as germination percentage. After recording the germination success, seedlings in each bag were thinned to single while discarding the other four.

The growth experiment with daily monitoring was continued for 15 weeks, during which all the bags were irrigated with tap water. After 100 days of growth, five plants from each treatment were randomly harvested for the determination of shoot length, root length, plant biomass, number of leaves, stem diameter, and number of branches. The harvested plants were washed with tap water to remove the soil adhering to their roots, wiped, and weighed. They were then oven dried at 105 °C to a constant weight, to calculate their dry weight. Flowering was assessed in terms of number of days to the appearance of the first flower, and the total flowers emerging per plant. The fruits (pods) were harvested at each alternative days and the yield was assessed in terms of number and weight of pods harvested per plant. Further the average length (cm) and diameter (mm) of the pods were also recorded. The chlorophyll and carotenoid contents of the leaves were estimated by following the procedure of Moran and Porath (1980) using *N*, *N*-dimethyl formamide (DMF) as an extractant. The optical density of the extract was read at 470, 647, and 664 nm in a UV–Visible spectrophotometer, and the concentration of pigments was determined as detailed by Wellburn (1994). The fruits (pods) of the ladies finger were analyzed for protein, carbohydrate, and mineral content by Kjeldahl, Anthrone, and dry ashing methods, respectively (Nielsen 2010). The total solid content was determined by heating the pods at 105 °C to a constant weight.

In the course of the experiment the plants were infested with leaf miners and leaf spot disease. The leaf miner infection, traced to *Liriomyza* spp. was seen in the symptoms of feeding punctures and leaf mines appearing as white speckles on the upper leaf surface (Ahmed 2000). Plants were considered infected by the fungus, *Alternaria alternate* when there were light brown spots on leaves, which later turned into concentric dark brown spots (Cho and Moon 1980; Werner 1987; Tohyama et al. 2005; Arain et al. 2012). When the intensity of the infection was particularly severe, the infected leaves become brown, eventually dying and falling off (Canihos et al. 1999; Amenduini et al. 2003; Antonijevic et al. 2007). There was also borer infestation in the fruits due to the *Earias vittella* (Sharma et al. 2010; Halder et al. 2015); it was quantified as weight percentage of the infected fruits to the total weight of fruits per treatment.

The data were statistically analyzed for assessing the extent of significance in the observed variations—especially by one-way analysis of variance and least significant difference (LSD)—as per standardized protocols (Alan and David 2001; Field 2009).

## Results and discussion

The substitution of the soil with parthenium vermicompost enabled significantly greater germination success of ladies finger seeds in comparison to controls (Table 1). In comparison with the controls (62.29%), a germination success of 85.71% was achieved with vermicompost treatment 5 t/ha followed by 81.71 and 77.14% with 3.75 and 2.5 t/ha, respectively. The increase in seed germination in vermicompost-amended soils may be due to the increased concentrations of nitrate and ammonium in them, relative to the control soils. It is now beyond the dispute that nitrate and ammonium are efficient breakers of seed dormancy, facilitating germination (Bewley and Black 1982; Hilhorst and Karssen 2000). In recent studies, Hussain et al. 2016a reported that parthenium vermicompost significantly enhanced the relative concentrations of nitrate and ammonium in soil compared with the controls.

**Table 1:** Seed germination, plant growth, flowering, and disease incidence in ladies finger plants grown in soil fortified with different concentrations of parthenium vermicompost

Parameters, average value	Vermicompost concentration				*F* value
	0 t/ha (control)	2.5 t/ha	3.75 t/ha	5 t/ha	
Germination					
Germination percentage	62.29 ± 6.46^a^	77.14 ± 7.10^b^	81.71 ± 7.47^c^	85.71 ± 9.17^d^	63.253*
Growth					
Shoot length (cm)	23.6 ± 2.97^a^	86.0 ± 6.86^b^	98.8 ± 7.98^c^	122.6 ± 10.21^d^	159.515*
Root length (cm)	34.4 ± 3.21^a^	41.4 ± 3.85^bc^	46.0 ± 4.58^c^	53.0 ± 5.24^d^	16.627*
Shoot diameter (mm)	4.48 ± 0.38^a^	10.22 ± 1.00^bc^	10.78 ± 0.89^c^	13.38 ± 1.30^d^	77.381*
Shoot dry weight (g)	1.96 ± 0.31^a^	17.26 ± 0.83^b^	26.00 ± 2.48^c^	30.67 ± 3.06^d^	195.511*
Root dry weight (g)	1.39 ± 0.13^a^	4.94 ± 0.47^b^	6.12 ± 0.59^c^	9.38 ± 0.93^d^	149.649*
No of leaves	11.8 ± 1.10^a^	24.8 ± 2.49^b^	28.4 ± 2.41^cd^	30.6 ± 2.97^d^	64.352*
No of branches	0.0 ± 0.00^a^	2.4 ± 0.55^b^	4.4 ± 0.89 ^cd^	5.2 ± 0.84^d^	59.852*
Flowering					
Days to first flowering	52.70 ± 4.85^a^	41.00 ± 2.91^bc^	39.10 ± 3.31^cd^	36.80 ± 1.93^d^	42.882*
Number of flowers per plant	2.90 ± 0.32^a^	7.80 ± 1.14^b^	11.80 ± 1.14^c^	12.90 ± 0.99^d^	224.036*
Diseases incidence					
Diseases incidence percentage	21.43 ± 4.95^a^	13.57 ± 4.29^bcd^	12.14 ± 4.88^cd^	12.14 ± 6.34^d^	2.976^n.s^

Results which do not differ significantly (LSD test; *p* < 0.05) carry at least one character in the superscript which is common

*n.s* not significant

* p < 0.05

**Fig. 1:**
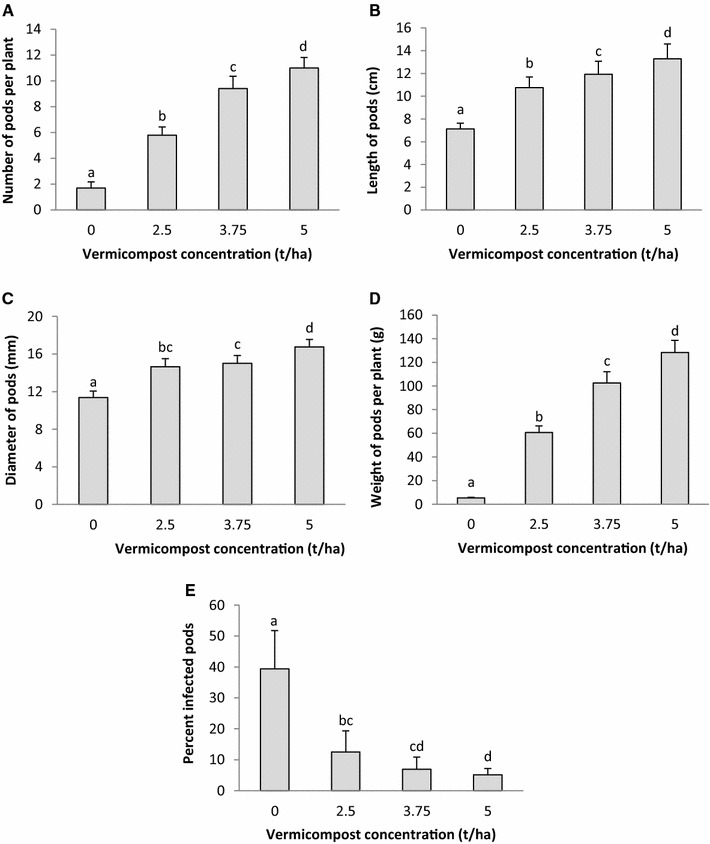
Effect of parthenium vermicompost on **A** number of pods, **B** length of pods, **C** diameter of pods, **D** weight of pods per plant, and **E** weight of infected pods of ladies finger. The standard deviation is indicated on the chart. Results which do not differ significantly (LSD test; *p* < 0.05) carry at least one character in the superscript which is common

There was also better growth of ladies finger in terms of all the variables studied compared with the controls (Table 1). An increase in growth was observed with increasing concentration of vermicompost in the soil, the trend being control <2.5 < 3.75 < 5 t/ha. The growth of ladies finger had shown significant enhancement even with a relatively small concentration of parthenium vermicompost (2.5 t/ha) in the container medium. Maximum shoot length 122.6 ± 10.21 cm, root length 53.0 ± 5.24 cm, shoot diameter 13.38 ± 1.30 mm, shoot dry weight 30.67 ± 3.06 g, root dry weight 9.38 ± 0.93 g, number of leaves 30.6 ± 2.97, and number of branches 5.2 ± 0.84 were recorded in plants grown in soil amended with 5 t/ha VC treatments. Parthenium vermicompost also induced early flowering and significantly higher number of flowers in ladies finger plants, in comparison to controls. The yield, in terms of number and weight of pods per plant, and the length and diameter of the pods, was also significantly higher in VC treatments than the controls (Fig. 1). Past studies have demonstrated that vermicompost derived from animal manure increased the growth and yield of several plant species (Doan et al. 2013; Joshi et al. 2015; Ayyobi et al. 2014; Xu et al. 2014; Akhzari et al. 2015; Kumar et al. 2015; Saxena et al. 2015).

Vermicompost derived from different substrates especially from animal manures are known to contain all the necessary plant nutrients in more bioavailable form than is present in the parent substrate (Edwards et al. 2011). It also contains diverse microflora, which are beneficial for soil health and the plant growth. A number of studies also reported the presence of plant growth regulators especially humic and fulvic acids, and phytohormones in manure-based vermicompost (Muscolo et al. 1999; Atiyeh et al. 2000, 2001, 2002; Arancon et al. 2005, 2008; Ievinsh 2011). It is the combined action of bioavailable nutrients, plant growth regulators, and soil microflora in the vermicompost that is responsible for enhancing the plant growth and yield (Chan and Griffiths 1988; Edwards and Burrows 1988; Wilson and Carlile 1989; Atiyeh et al. 1999; Ayyobi et al. 2014; Xu et al. 2014; Akhzari et al. 2015; Kumar et al. 2015; Saxena et al. 2015). In the present study we suggest that parthenium vermicompost may have also imbibed with similar attributes to that of the manure-based vermicomposts that has resulted in greater germination success, better plant growth, and yield of ladies finger plants. Recently (Hussain et al. 2016a, b) have reported that parthenium vermicompost contains a number of fatty acids, alcohols, alkanes, alkenes, and nitrogenous compounds in it and enhanced the microbial biomass carbon of the soil. Beside these factors, parthenium vermicompost, as like manure-based vermicomposts, is also known to induce positive impact on soil physical properties and hence may also have contributed to the better plant growth (Hussain et al. 2016a).

**Fig. 2:**
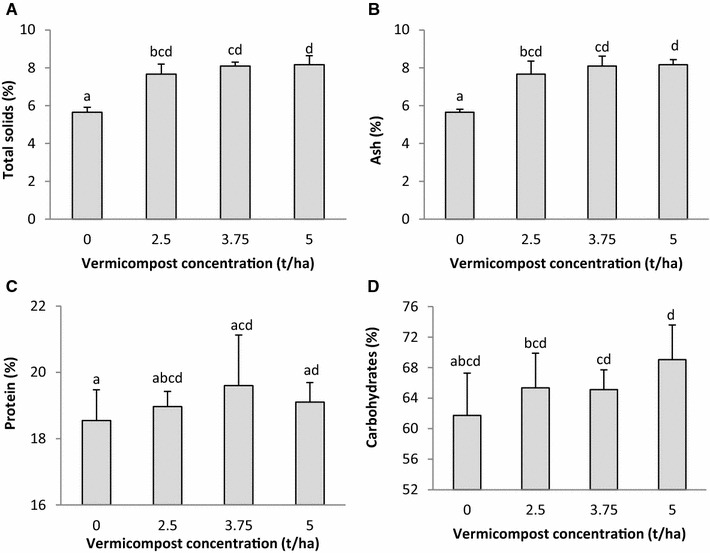
Effect of parthenium vermicompost on **A** totals solids, **B** ash content, **C** protein content, and **D** carbohydrate content of ladies finger pods. The standard deviation is indicated on the chart. Results which do not differ significantly (LSD test; *p* < 0.05) carry at least one character in the superscript which is common

The levels of pigments in the leaves of the ladies finger plants were significantly influenced by the vermicompost application (Table 2). Maximum chlorophyll (1.43 ± 0.09 mg/g) and carotenoid (0.90 ± 0.07 mg/g) content was recorded in 5 t/ha vermicompost treatments. The total solids and ash (mineral) content of vermicompost-treated plants were also significantly higher compared to the controls. An increase in the protein and carbohydrate concentrations were also recorded in the plants grown in vermicompost-amended soils (Fig. 2). All these gains are perhaps due to the greater bioavailability of nutrients in vermicompost treatments compared to the controls, as has been earlier seen with manure-based vermicompost (Abduli et al. 2013; Ayyobi et al. 2014; Akhzari et al. 2015; Yadav et al. 2015).

**Table 2:** Chlorophyll and carotenoid content of ladies finger plants grown in soil fortified with different concentrations of parthenium vermicompost

Parameters, average value	Vermicompost concentration				*F* value
	0 t/ha (control)	2.5 t/ha	3.75 t/ha	5 t/ha	
Chlorophyll ‘a’ (mg/g)	0.47 ± 0.04^a^	0.62 ± 0.04^bc^	0.63 ± 0.04^c^	0.91 ± 0.07^d^	68.604*
Chlorophyll ‘b’ (mg/g)	0.25 ± 0.02^ac^	0.29 ± 0.02^bc^	0.26 ± 0.01^c^	0.52 ± 0.04^d^	120.380*
Total Chlorophyll (mg/g)	0.72 ± 0.04^a^	0.90 ± 0.03^bc^	0.89 ± 0.04^c^	1.43 ± 0.09^d^	149.604*
Carotenoid (mg/g)	0.30 ± 0.03^a^	0.43 ± 0.04^bc^	0.40 ± 0.04^c^	0.90 ± 0.07^d^	154.545*

Results which do not differ significantly (LSD test; *p* < 0.05) carry at least one character in the superscript which is common

* *p* < 0

Parthenium vermicompost was effective in inducing resistance in the ladies finger plants against pests and pathogens. A significant reduction in the infestation of leaf miners, leaf spot disease, and fruit borers was observed (Table 1; Fig. 1E), the trend being 0 t/ha (control) >2.5 > 3.75 > 5 t/ha. At the early stages of growth the seedlings were seen severely infested by leaf liner; however, as the growth increased the number of incidents decreased in proportion. Previous studies have reported that plants grown in soil amended with manure-based vermicompost have shown a reduction in the pest and disease attack (Edwards et al. 2010; Cardoza and Buhler 2012). Different authors have provided different explanations for the pesticidal properties of the vermicompost which basically revolve around two conjectures: better nutrient availability hence greater vitality in warding off infection, and presence of pathogen-destroying microorganisms (Arancon et al. 2005; 2008; Yardim et al. 2006; Cardoza 2011; Singh et al. 2013; Xiao et al. 2016). The present studies indicate that the vermicompost of parthenium is also imbibed with a similar attribute. Past studies on manure-based vermicompost have indicated that better nutrient availability and presence of antimicrobial compounds such as flavonoids, phenolics, and humic acids in the vermicompost may have induced the resistance to pathogens in the plants (Graham and Webb 1991; Hill et al. 1999; Haviola et al. 2007; Sahni et al. 2008; Edwards et al. 2010). Similarly beneficial attributes seem to be present in parthenium’s vermicompost as well.

## Conclusion

In a field study, effect of vermicompost produced solely from an allelopathic weed parthenium has been investigated on germination, growth, yield, and quality of ladies finger (*Abelmoschus esculentus*). The effect of the vermicompost in inducing resistance in ladies finger against disease was also assessed. In general vermicompost application increased germination success, plant growth, and yield—the positive effect increased in prominence as the extent of vermicompost application was enhanced from 2.5 to 5 t/ha. Parthenium vermicompost also induced beneficial changes in the biochemical and mineral content of the ladies finger. Additionally, ipomoea vermicompost induced resistance in ladies finger towards disease and pest attacks. Overall, contrary to the toxic and allelopathic nature of parthenium, its vermicompost manifests the attributes of highly plant-friendly organic fertilizer that vermicomposts derived from animal manure are known to possess.
